# Actions of *Huangqi decoction* against rat liver fibrosis: a gene expression profiling analysis

**DOI:** 10.1186/s13020-015-0066-5

**Published:** 2015-12-18

**Authors:** Gui-biao Zhang, Ya-nan Song, Qi-long Chen, Shu Dong, Yi-yu Lu, Ming-yu Su, Ping Liu, Shi-bing Su

**Affiliations:** Research Center for Traditional Chinese Medicine Complexity System, Shanghai University of Traditional Chinese Medicine, Shanghai, 201203 China; Liver Disease Institute, Shuguang Hospital, Shanghai University of Traditional Chinese Medicine, Shanghai, 201203 China

## Abstract

**Background:**

*Huangqi decoction* (HQD) is used for liver fibrosis and cirrhosis treatment in Chinese medicine. This study aims to investigate the pharmacological actions of HQD against liver fibrosis in rats by high-throughput gene expression profiling, network analysis and real-time qRT-PCR.

**Methods:**

We analyzed the profiles of differentially expressed genes (DEGs) in dimethylnitrosamine (DMN)-induced liver fibrosis in rat. The liver tissue samples of control group (n = 3), model group (n = 3) and HQD group (n = 3) were examined by microarrays. Pathways were analyzed by KEGG. Pathway-gene and protein–protein interaction (PPI) networks were constructed with Cytoscape software. The expression of candidate genes was verified by qRT-PCR. *P* values less than 0.05 indicated statistical significance.

**Results:**

Collagen deposition and hydroxyproline (Hyp) content were decreased in the HQD group compared with the model group (*P* < 0.001), while that of Hyp in the model group were increased compared with the control group (*P* < 0.001). In comparison with the model group, 1085 DEGs (all *P* < 0.05, |fold change| >1.5) and 52 pathways in the HQD group were identified. TGF-beta, ECM-receptor interaction, and the cell adhesion molecules pathways were significantly recovered by HQD (*P* < 0.001). A pathway-gene network was constructed, including 303 DEGs and 52 pathways, and 514 nodes and 2602 edges, among 142 genes with node degrees greater than 10. The expressions of PDGFra, PDGFrb, PDGFb, PDGFd, COL1A1, COL1A2, COL5A2, and THBS1 were significantly down-regulated by HQD (*P* < 0.001).

**Conclusion:**

HQD down-regulated the expressions of PDGFra, PDGFrb, PDGFb, PDGFd, COL1A1, COL1A2, COL5A2 and THBS1, and TGF-β and PDGF signaling pathways in the DMN-induced liver fibrosis in rats.

**Electronic supplementary material:**

The online version of this article (doi:10.1186/s13020-015-0066-5) contains supplementary material, which is available to authorized users.

## Background

Liver fibrosis and cirrhosis were found associated with sustained wound-healing responses to chronic liver injury caused by viral, autoimmune, drug-induced, cholestatic, alcoholic, or metabolic pathogenesis [1]. However, the matrix components of scar tissue in cirrhosis with different etiologies were similar [2]. As the extracellular matrix (ECM) undergoes continuous remodeling, with the production and degradation of ECM tissues [1, 3], initial fibrosis was considered reversible, although some previous studies did not support this view [1, 2]. Moreover, fibrosis stage information not only indicates cirrhosis development but also evaluates treatment response [4]. Despite the high incidence of hepatic fibrosis worldwide, there is currently no validated anti-fibrogenic therapy.

In Chinese medicine (CM), a formula targets many molecules in the cells to exhibit therapeutic efficacy and reduce adverse effects [5–7]. *Huangqi decoction* (HQD) is one of the several CM formulae that can improve liver function and quality of life in patients with liver disorders [6, 7]. HQD consists of two medicinal herbs, *Radix Astragali* (*Huang Qi*) and *Radix et Rhizoma Glycyrrhizae* (*Gan Cao*), mixed in a 6:1 (w/w) ratio [8]. In laboratory studies on rats, HQD exerted significant therapeutic effects on liver fibrosis or cirrhosis induced by dimethylnitrosamine (DMN) [8, 9] and bile duct ligation [10].

Microarray analysis can identify potential disease biomarkers [11, 12]. Network-based analyses like network pharmacology can systematically reveal complex biological relationships [13–15]. As there are complex interactions among multiple compounds, targets, and signal pathways, gene expression profiling [16] and network pharmacological methods [17] reveal some pharmacological actions of CM formulae. Microarray-based network analysis can be used to identify potential drug targets and biomarkers [18] and reveal some mechanisms of CM formulae [19]. This study aims to investigate the pharmacological actions of HQD against liver fibrosis in rats with gene expression profiling, network analysis and real-time qRT-PCR.

## Methods

### Materials

DMN was purchased from Sigma-Aldrich (St. Louis, MO, USA). *R. Astragali* (30 g) and *R. Glycyrrhizae* (5 g), were provided by Shanghai Huayu Herbs Co. Ltd. (Shanghai, China). The herbs of HQD were accredited by pharmacologists and High Performance Liquid Chromatography (HPLC) as previously reported [10], and prepared by Shanghai Shuguang Hospital. The preparation of aqueous extracts of the herbs and quality control were carried out as described previously [8–10]. The medicinal herb mixture was extracted in boiling water, and the aqueous extracts were vacuum-dried at 60 °C to obtain a powder, and then stored at −20 °C. The extract was prepared by a standardized process and strict quality control according to the guidelines of the Chinese State Food and Drug Administration [9].

### Animal experiments

Similar to previous studies [8, 9], 40 male Wistar rats (180–200 g) were housed in standard animal conditions with controlled temperature (17–25 °C), humidity (45–60 %), and a 12-h/12-h light/dark cycle. They were arbitrarily allocated into two groups: a control group (n = 10) and a DMN-treated group (n = 30). DMN was administered intraperitoneally at 10 mg/kg for 3 consecutive days each week for 4 weeks in the DMN-treated group; control rats received equal quantities of physiological saline in the same way. At the end of the second week, three and six rats from the control and DMN-treated groups, respectively, were dissected for fibrosis development assessment. The remaining DMN-treated rats were further arbitrarily allocated into two groups: a model group with saline treatment (n = 12) and an HQD-treated group (n = 12). In addition to continuous DMN treatment, the rats received daily administration of saline or HQD given intragastrically at 1 mL/100 g. At the end of the fourth week, all rats were sacrificed and liver tissue samples were collected. All procedures were carried out in accordance with the “Regulations for the administration of affairs concerning experimental animals,” published in 1988 by the State Scientific and Technological Commission [20]. Shanghai University of Traditional Chinese Medicine’s Animal Ethics Committee approved this study protocol (No. 2011009, Additional file 1) before its conduct.

### Histological evaluation and hepatic hydroxyproline (Hyp) assay

Liver specimens were preserved in 4 % paraformaldehyde, dehydrated in a graded alcohol series, embedded in paraffin blocks, sectioned to 5-μm-thick slices, placed on glass slides, and stained with H&E and Sirius Red. Fibrosis scores were determined after examination of three different areas of the tissue slide from each rat. Fibrosis was graded according to Scheuer’s method [21] as follows: grade 0, normal liver; grade 1, increased collagen without formation of septa (small satellite expansion of portal fields); grade 2, formation of incomplete noninterconnecting septa, from portal tract to central vein; grade 3, complete but thin interconnecting septa, dividing the parenchyma into separate fragments; and grade 4, complete cirrhosis, similar to grade 3 but with thicker septa.

Liver tissues (100 mg) were prepared for Hyp determination according to Jamall et al. [22], using a Hydroxyproline kit (Nanjing Jiancheng Bioengineering Institute, Nanjing, China). Hyp liver content expressed as μg/g wet weight, indirectly indicated the tissue collagen content.

### cDNA microarray detection and data analysis

Total RNA was extracted by TRIzol Reagent (Invitrogen, Carlsbad, CA, USA) following the manufacturer’s instructions and checked for a RIN number to inspect RNA integration by an Agilent Bioanalyzer 2100. Qualified total RNA was purified by the RNeasy mini kit and RNase-Free DNase Set (QIAGEN, GmBH, Germany). Total RNA was amplified and labeled by the Low Input Quick Amp Labeling Kit, One-Color (Agilent Technologies, Santa Clara, USA), under the manufacturer’s instructions. Labeled cRNA was purified by the RNeasy mini kit. Each slide was hybridized with 1.65 μg of Cy3-labeled cRNA by a Gene Expression Hybridization Kit (Agilent technologies, Santa Clara, CA, US) in a hybridization oven, according to the manufacturer’s instructions. After 17 h of hybridization, slides were washed in staining dishes (Thermo Shandon, Waltham, USA) with a Gene Expression Wash Buffer Kit (Agilent Technologies), under the manufacturer’s instructions. Slides were scanned by an Agilent Microarray Scanner G2565BA (Agilent Technologies) with default settings, Dye channel: Green, Scan resolution = 5 μm, PMT 100 %, 10 %, 16 bit. Raw data were normalized by Gene Spring Software 11.0 (Agilent Technologies). The differences among samples, including DEG, hierarchical clustering, Gene Ontology (Go), and signalling pathways were calculated by the SAS system (Shanghai Biochip, Shanghai, China). Based on the data of protein–protein interactions from the HPRD [23] and STRING [24] databases, pathway-gene and gene PPI networks were constructed by Cytoscape 2.8.3 (http://www.cytoscape.org) [25], and R^2^ of PPI networks was also constructed by Cytoscape, which represents part of how much information can be explained by the independent variables in the dependent variable.

### Quantitative real-time polymerase chain reaction (qRT-PCR) analysis

Total RNA was converted to cDNA by the Moloney murine leukemia virus (M-MLV, Life, USA) for 1 h at 37 °C. We used oligonucleotide primer pairs (SBS Genetech Co., Ltd, Shanghai, China.) selected and tested for their specificity, including PDGFra, PDGFrb, PDGFb, PDGFd, COL1A1, COL1A2, COL5A2, ITGA5, THBS1, and IL1R1 mRNAs (Table 1), and carried out a qRT-PCR analysis by a SYBR green chemistry kit (TOYOBO, Osaka, Japan). Each experiment was performed in triplicate with β-actin as an endogenous control. Each gene was quantified relative to the calibrator. The PCR program consisted of an initial period of 10 min at 95 °C followed by 40 thermal cycles, each of 10 s at 95 °C, 5 s at 65 °C, and 30 s at 70 °C by an ABI 9600 real-time PCR system (ABI, USA). The data obtained were analyzed by the $2^{{ - \varDelta \varDelta {\text{C}}_{\text{T}} }}$ method, and were normalized according to the β-actin expression level. Melting curves for each PCR reaction were generated to ensure the purity of the amplification products.

**Table 1:** Primer sequences for RT-PCR

Gene	Description	Primer sequences	
		Forward	Reverse
PDGFra	Platelet derived growth factor receptor, alpha polypeptide	GAAGGTGGTTGAAGGAACAGC	AGGCTCCCAGCAAGTTTACAA
PDGFrb	Platelet derived growth factor receptor, beta polypeptide	CGCGTGCGTCTGTTTTCAATTT	GCGTGGGCTGTGGAATTTCTAA
PDGFb	Platelet derived growth factor beta polypeptide	GGCCTTCTTAAAGATTGGCTTCT	GCCTCATAGACCGCACCAAC
PDGFd	platelet derived growth factor D	CCCATTCGGAGGAAGAGAAG	ATCAGGAAGTTGGCGGACG
COL1A1	Collagen, type I, alpha 1	ACGTCCTGGTGAAGTTGGTC	ACCAGGGAAGCCTCTCTCTC
COL1A2	Collagen, type I, alpha 2	GGAGGGAACGGTCCACGAT	GAGTCCGCGTATCCACAA
COL5A2	Collagen, type V, alpha 2	GGAAATGTGGGCAAGACTGT	TTGATGGTGGTGCTCATTGT
THBS1	Thrombospondin 1	AACGTGGATCAGAGGGACAC	GTCATCGTCATGGTCACAGG
ITGA5	Integrin, alpha 5 (fibronectin receptor, alpha polypeptide)	GTCGGGGGCTTCAACTTAGAC	CCTGGCTGGCTGGTATTAGC
IL1R1	Interleukin 1 receptor, type I	AGAGGAAAACAAACCCACAAGG	CTGGCCGGTGACATTACAGAT
β-actin	Actin, beta	TCCTGTGGCATCCACGAAACT	GAAGCATTTGCGGTGGACGAT

### Statistical analysis

The result was expressed as mean ± standard deviation (SD). Statistical analysis was performed by SPSS software (SPSS, Chicago, USA). The statistical significance of differences between two groups was analyzed by the Student’s unpaired *t* test or among multiple groups by the one-way analysis of variance (ANOVA), followed by LDS test. In all analyses, *P* values less than 0.05 indicated statistical significance.

## Results

### Histological changes

**Fig. 1:**
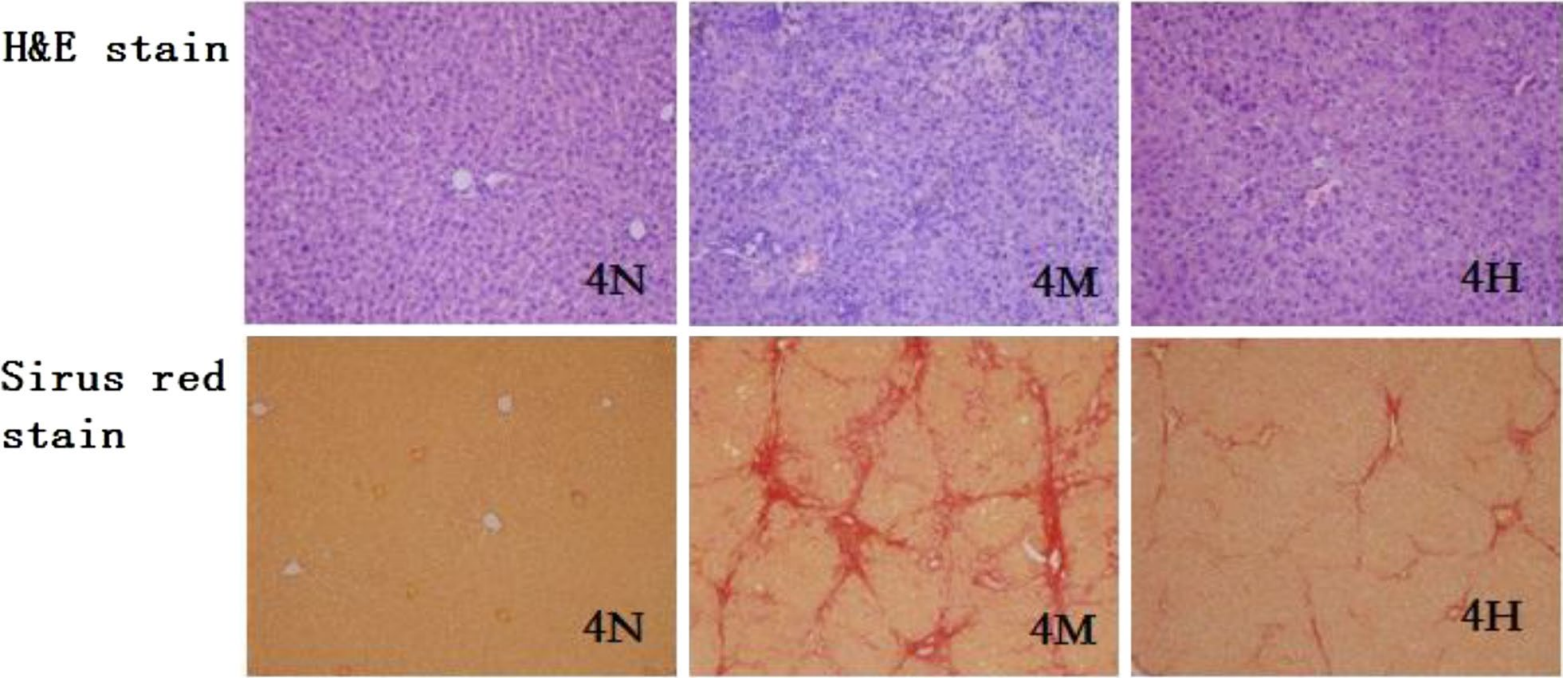
Effects of HQD on histological changes. N, control group; M, model staining (original magnification ×100)

H&E stain showed no morphological abnormality in the control rats (Fig. 1). Persistent reduction of hepatocytes, gradual proliferation of cholangiocytes, and continuous infiltration of neutrophils were noted in the DMN model group. These observation indices were better in the HQD- treated group compared with the model group. The Sirius Red staining results showed that in the control group, there was little collagen except around the small central venous walls. In the model group at 4 weeks, collagen was stretched from the portal area to lobular areas, and incomplete septa were also observed. Cirrhotic nodules had formed in the model group; by comparison, collagen deposition had decreased in the HQD-treated group (Fig. 1). Liver Hyp content and changes in the collagen hyperplasia are shown in Table 2. After 4 weeks of DMN injection, the model group rats’ liver Hyp content increased significantly (*P* < 0.001) and was 2.47 times as much as that in the control group; the degree of collagen hyperplasia in the model group was mainly concentrated in stages III and IV. Compared with the model group, Hyp content in the HQD-treated group decreased significantly (*P* < 0.001) and was 0.43 times as much as that in the control group; the degree of collagen hyperplasia in the model group was mainly concentrated in stages II and III.

**Table 2:** Effects of HQD on fibrotic grades and Hyp content

Group	n	Hyp content (μg/g wet liver)	Fibrotic grade				
			0	I	II	III	IV
Control	9	181.78 ± 43.45**	9	0	0	0	0
Model	9	427.90 ± 129.46	0	0	1	5	3
HQD-treated	9	245.86 ± 40.85**	0	1	5	3	0

*Grade 0* normal, *grade 1* very slight, *grade 2* slight, *grade 3* moderate, *grade 4* severe. Data are expressed as numbers of animals with specific fibrotic grades

** *P* < 0.01, vs. model group

### Gene expression data analysis

**Fig. 2:**
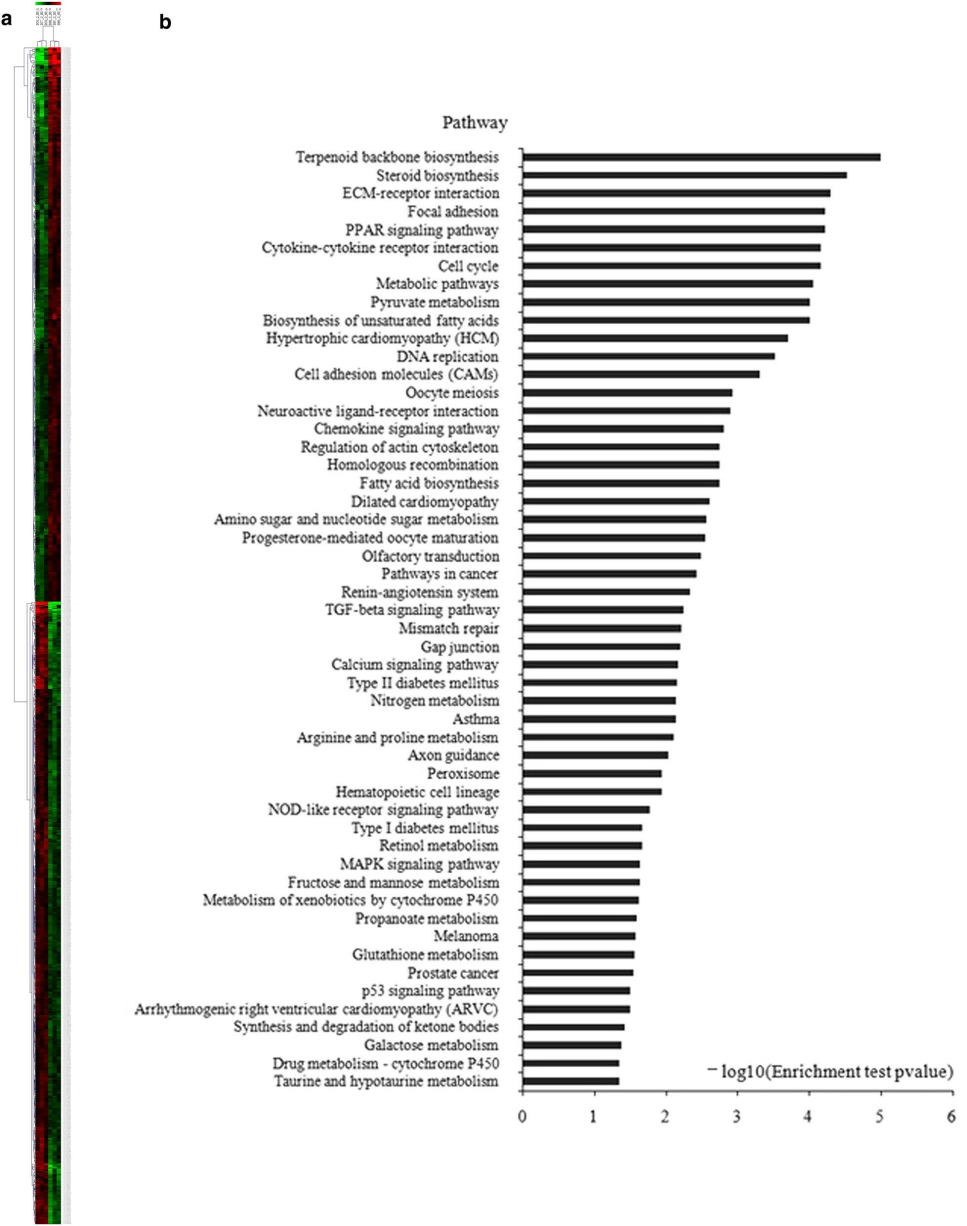
Hierarchical clustering and pathway enrichment of DEGs (n = 3). **a** DEGs of three HQD-treated vs. three DMN-treated rats; an unsupervised hierarchical clustering of DEGs between HQD-treated and DMN-treated rats showing significantly differential expression revealed two distinct clusters. **b** Fifty-two enriched pathways based on DEGs showing a difference between HQD-treated and DMN-treated rats (*P* < 0.05). *Red nodes* represent genes, *blue nodes* represent pathways, and larger *circles* represent higher degrees

We identified differentially expressed genes (DEGs) in the microarray data, followed by pathway enrichment analysis. We profiled genome-wide gene expression for six liver fibrosis rats (three rats in the model and three in the HQD-treated group). Compared with the model group, the expression of 1085 genes in the HQD-treated group was significantly changed (all *P* < 0.05, fold change >1.5 or <0.67); among them, 518 genes were up-regulated and 567 were down-regulated (Fig. 2a). Pathway enrichment analysis of 1085 DEGs showed that these genes were significantly enhanced in 52 pathways (all *P* < 0.05; Fig. 2b).

The TGF-beta signaling pathway was enhanced by 8 DEGs (Table 3); TGF-beta was involved in liver fibrosis [26]. The ECM-receptor interaction pathway, the cell adhesion molecules (CAMs) pathway, and the focal adhesion pathway were involved in the development of fibrosis lesions [27, 28].

**Table 3:** KEGG pathway annotations of Dif-gene enrichment

KEGG pathway	Count	Enrichment test *P* value	Gene
TGF-beta signaling pathway	8	0.0057	THBS1, AMHR2, INHBC, FST, DCN, LTBP1
ECM-receptor interaction	17	0.00005	ITGA5, COL1A1, THBS1, COL1A2, COL5A2
Cell adhesion molecules	14	0.0005	CLDN4, CLDN9, SELE, CLDN5, VCAM1
Focal adhesion	27	0.00006	ITGA5, THBS1, COL1A2, COL5A2, PDGFrb, PDGFb, PDGFd

### Pathway-gene network analysis

**Fig. 3:**
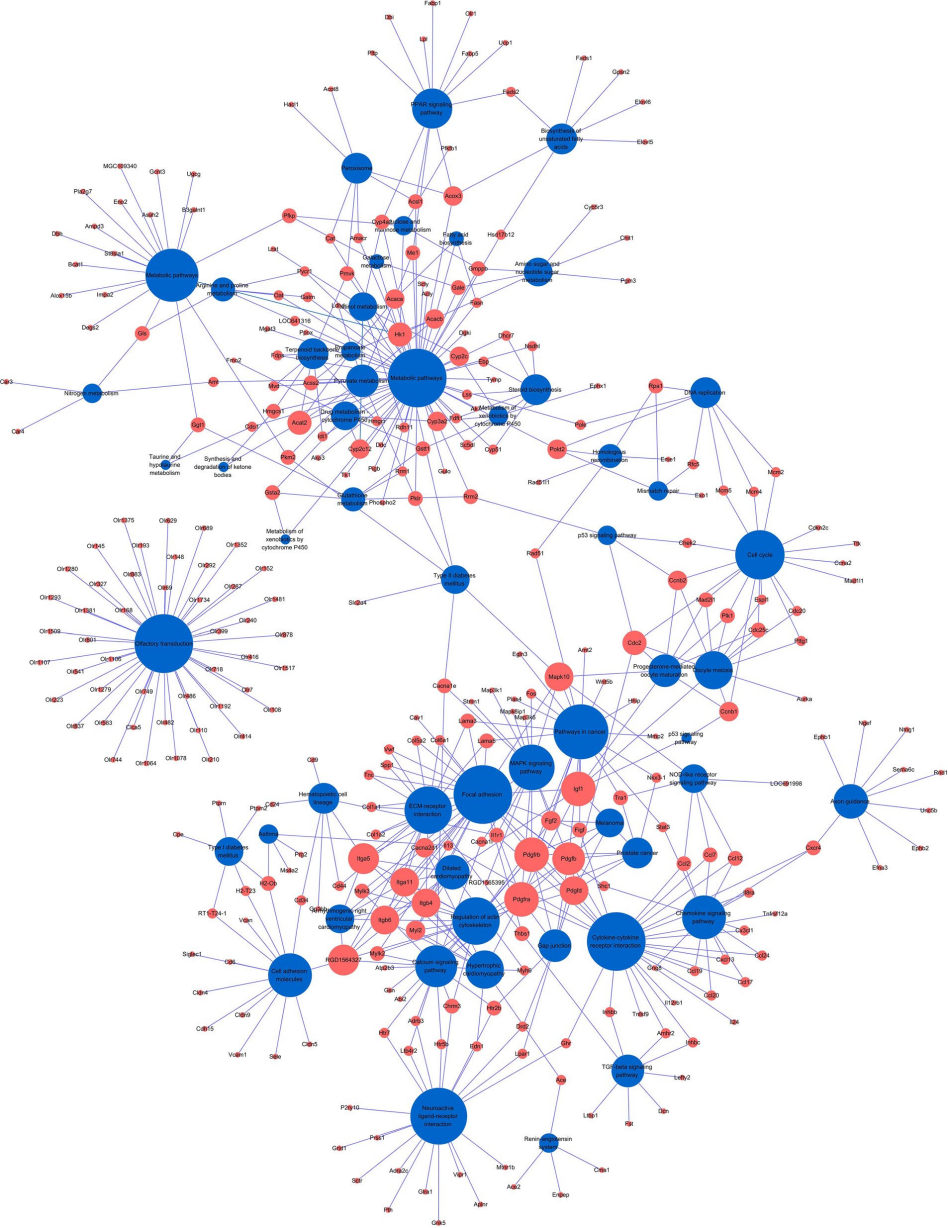
Pathway-gene network visualized by Cytoscape. Interaction network analysis of 303 genes. The 303 altered genes were connected in a network based on signaling pathways. *Blue* signaling pathway; Red, DEGs. PDGFra, PDGFrb, PDGFb, PDGFd, COL1A1, COL1A2, COL5A2, ITGA5, THBS1, and IL1R1 genes had the highest degree values; therefore, they might be important in liver fibrosis pathogenesis

We constructed a pathway-gene network based on 52 enrichment pathway-related genes by Cytoscape 2.8.3 to identify key genes in the pathway. The pathway-gene network contained 355 nodes and 562 edges, including 303 genes and 52 pathways (Fig. 3).

### Protein–protein interaction (PPI) network analysis

**Fig. 4:**
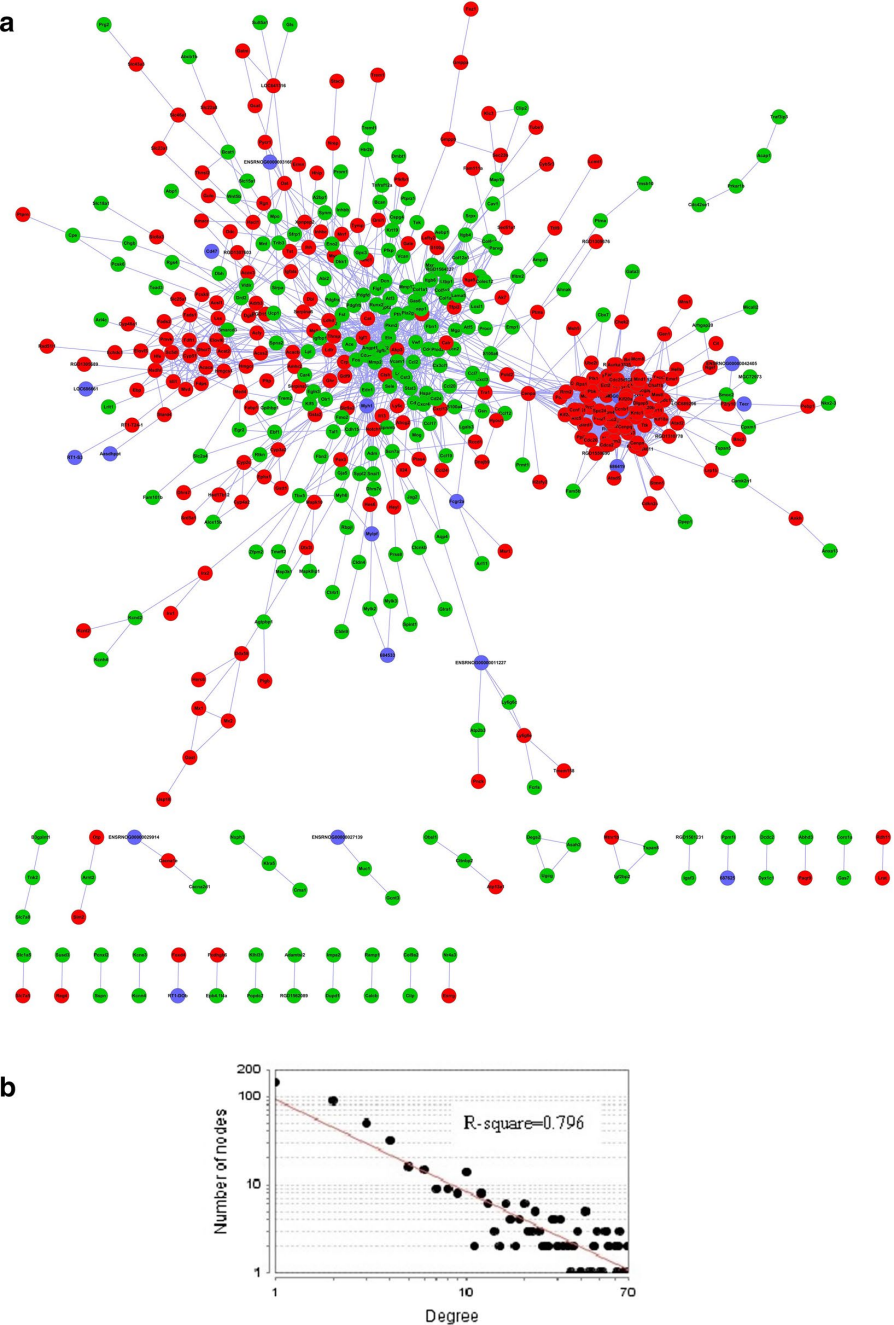
DEG PPI network. **a** PPI network visualized by Cytoscape. *Red nodes* represent up-regulated DEGs, *green nodes* represent down-regulated DEGs, *purple nodes* represent connect genes, and each edge represents the interaction between them. **b** The degree of nodes in the PPI network followed a power law distribution, indicating that the PPI network is scale-free

We constructed a DEG PPI network (Fig. 4a) by Cytoscape 2.8.3, to find key points (i.e., the main potential drug targets). The DEG PPI network contained 514 nodes and 2602 edges, including 142 genes with node degree >10. The DEG PPI network centralization and heterogeneity were 0.115 and 1.502, respectively. As most biological networks are scale-free, the node degrees follow the power law distribution rather than the Poisson distribution [29], we also tested whether the MI network was scale-free like other biological networks. The node degree distribution of the two PPI networks (R^2^ = 0.796, *P* < 0.001), indicating that the PPI network was scale-free (Fig. 4b).

Degree and betweenness were analyzed for the networks to specify the importance of a certain node and how this node influences the communication between the other two nodes. The degree of a node (biomolecule) is the number of targets that the drug has (respectively the number of drugs targeting the protein). The betweenness of a node is defined as the ratio of the number of shortest paths passing through a node to the total number of paths passing through the node [30].

### Validation of microarray results by qRT-PCR analysis

The microarray results were verified by a qRT-PCR analysis using the identical RNA samples. Ten DEGs including PDGFra, PDGFrb, PDGFb, PDGFd, COL1A1, COL1A2, COL5A2, ITGA5, THBS1 and IL1R1, closely related to liver fibrosis, were chosen for the real-time qRT-PCR analysis. Compared with the control group, PDGFra, PDGFrb, PDGFb, PDGFd, COL1A1, COL1A2, COL5A2 and THBS1 had significantly increased (*P* < 0.001) in the model group; however, IL1R1 had significantly decreased (*P* < 0.001). Compared with the model group, PDGFrb, COL1A1, and COL1A2 had significantly decreased (*P* < 0.001) in the HQD-treated group and PDGFra, PDGFb, PDGFd, COL5A2, and THBS1 had significantly decreased (*P* < 0.001), but there was no significant difference for IL1R1 and ITGA5. The results of the qRT-PCR analysis for these selected genes were consistent with that obtained from microarray data, although the fold changes in the expression level differed.

## Discussion

HQD has been used to treat consumptive diseases, restlessness, hydrodipsia, anorexia, and chronic liver diseases [3, 4]. In the present study, HQD alleviated liver fibrosis induced by DMN. Both Sirius Red staining and H&E staining results demonstrated a significant anti-fibrotic effect of HQD, confirmed by liver Hyp content, which was consistent with the anti-fibrotic effect of HQD in previous research [3, 31].

Recent research has focused increasingly on investigating drug efficacy by high-throughput technologies and network analysis [32–34]. Liver fibrosis development is associated with a network of profibrogenic and inflammatory signaling pathways [1]. In this study, we found that 518 up-regulated genes, 569 down-regulated genes, and 52 signaling pathways were regulated by HQD treatment, indicating that HQD ameliorated liver fibrosis lesions through regulating multiple genes and multiple signal pathways.

**Fig. 5:**
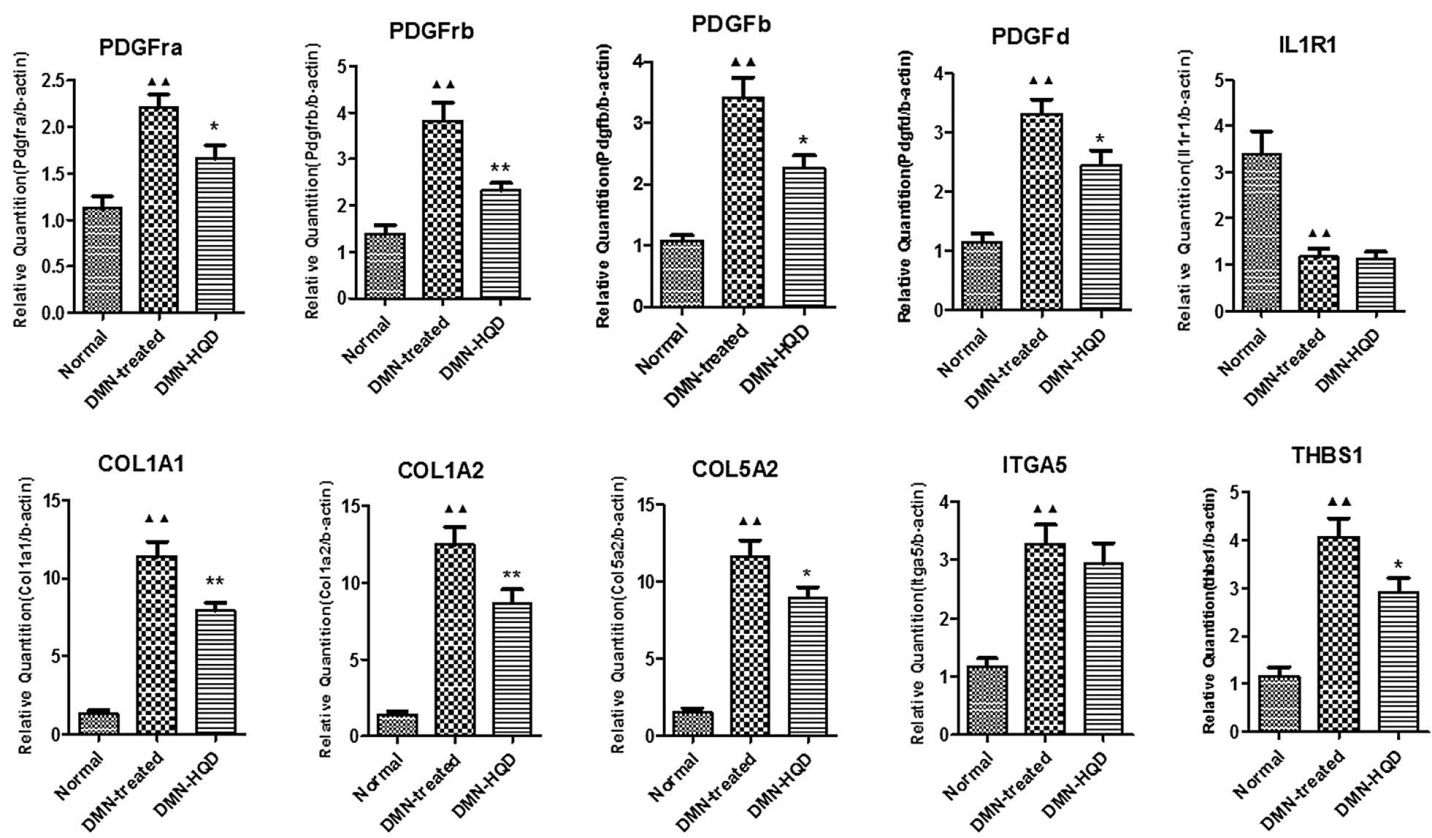
Validation of microarray data by qRT-PCR analysis (n = 9). Results were expressed as the relative quantification normalized to β-actin mRNA expression. ^▲▲^ *P* < 0.01 vs. normal group; *P < 0.05, **P < 0.01 vs. model group

Highly connected hubs in protein interaction networks are potential drug targets [35]. The highly connected hubs of both pathway-gene and PPI networks revealed 10 high degree genes (Table 4), and the levels of PDGFra, PDGFrb, PDGFb, PDGFd, COL1A1, COL1A2, COL5A2 and THBS1 expressions were significantly down-regulated by HQD treatment (Fig. 5), suggesting that these genes might be the potential molecular targets of HQD against liver fibrosis.

**Table 4:** Foldchange and degree of Dif-gene

Gene	Foldchange^a^	Degree of pathway-gene network	Degree of gene PPI network
PDGFra	−1.56	9	7
PDGFrb	−1.61	9	7
PDGFb	−1.62	8	17
PDGFd	−1.92	6	4
COL1A1	−1.77	2	24
COL1A2	−1.54	2	20
COL5A2	−1.52	2	18
ITGA5	+1.77	7	
THBS1	−2.22	3	
IL1R1	+1.58	3	

^a^Fold change referred to an average ratio for only these cases (frequency) in which the ratio of change (HQD-treated/DMN-treated) was over 1.5-fold. +, up-regulation; −, down-regulation, *P* < 0.05

Among the 52 enriched pathways, the TGF-β signaling pathway was the most prominent direct inducer of collagen transcription in HSC [36]. THSP1 is a complex multifunctional protein released from platelet α-granules [37, 38], and was involved in wound healing and fibrosis [39]. The latent TGF-β activation induced by THSP1 could prevent liver fibrosis [40]. ITGA5 regulated MMPs gene expression during the fibrosis process [41]. Additionally, fibronectin, via ITGA5 receptors, might up-regulate MMP-9 gene expression [41].

HSCs produce ECM molecules and release profibrogenic cytokines, including PDGFs and PDGFrs [42]. These cytokines are critical indicators in the pathogenesis of hepatic fibrosis [43]. PDGF signaling is one of the best characterized pathways of HSC activation [44]. Rapid induction of PDGFrb, is followed by development of a contractile, fibrogenic phenotype that correlates with the degree of fibrosis and inflammation [45]. PDGF induces activation of the downstream molecules Erk and Akt in activated HSCs, which is associated with cellular proliferation and migration [46], and transgenic over-expression of PDGF leads to liver fibrosis in mice [47]. PDGF action is determined by the relative expression of PDGFra and PDGFrb on the surface of myofibroblasts [48]. PDGF is the potent mitogen for HSCs, and its antagonism could be an anti-fibrotic strategy [49]. PDGF receptor tyrosine kinase inhibitors might be effective in the treatment of fibrotic diseases [42]. PDGFs and/or PDGFrs could be the target of HQD to slow down the process of liver fibrosis.

COL1A1 expression levels in transdifferentiated epithelial cells were at least one to two orders of magnitude lower than that in myofibroblasts [50]. COL1A1, COL3A1, COL5A1, and COL5A2 chains were induced significantly in active multiple sclerosis lesions and even more in inactive lesions. These chains interact to form collagen types I, III, and V, which are fibrillar collagens [51]. Suppressing COL1A1 and COL1A2 expressions might decrease the activation of HSCs and eventually prevent liver fibrosis [52].

Treatment with HQD altered the expression of a number of genes, including different PDGFs and COL1As, and also multiple pathways like the TGF-beta and PDGF signaling pathways, which would potential targets of HQD. Pathway enrichment analysis also showed that HQD can affect type 2 diabetes mellitus, hypertrophic cardiomyopathy, and other diseases [53]. All the major constituents of Astragalus significantly lowered high blood glucose levels and body weight and improved impaired glucose tolerance in type 2 diabetic models [53, 54], suggesting that HQD might treat multiple diseases through regulating multiple genes and multiple signal pathways.

## Conclusion

HQD down-regulated the expressions of PDGFra, PDGFrb, PDGFb, PDGFd, COL1A1, COL1A2, COL5A2 and THBS1, and TGF-β and PDGF signaling pathways in the DMN-induced liver fibrosis in rats.

## Additional file

10.1186/s13020-015-0066-5 The inspection of animal experimental ethical.

## Abbreviations

CM: Chinese medicine
HQD: *Huangqi decoction*
DEGs: differentially expression genes
DMN: dimethylnitrosamine
ECM: extracellular matrix
Hyp: hydroxyproline
HSCs: hepatic stellate cells
M-MLV: moloney murine leukemia virus
CAMs: cell adhesion molecules
MMPs: metalloproteinase
PDGFs: platelet-derived growth factors
PDGFrs: PDGF receptors
ITGA5: integrin alpha5
THSP1: thrombospondin 1
COL1A: collegen type 1 alpha
TGF-β: transforming growth factor-β
PPI: protein–protein interaction
qRT-PCR: quantitative real-time polymerase chain reaction
HPLC: High Performance Liquid Chromatography
SD: standard deviation
