# Supplementary material for: Actions of Huangqi decoction against rat liver fibrosis: a gene expression profiling analysis
Source: Chin Med. 2015 Dec 18;10:39. doi: 10.1186/s13020-015-0066-5 (PMC4683959; doi:10.1186/s13020-015-0066-5)
Supplement: Supplementary file 1 — 10.1186/s13020-015-0066-5 The inspection of animal experimental ethical. [file 13020_2015_66_MOESM1_ESM.pdf]

上海中医药大学动物实验伦理委员会

动物实验伦理审查表

登记编号：2011009

课题名称：黄芪汤及其成分抑制肝纤维化的系统生物学评价	专题负责人：张贵彪

简述动物实验方案（阐明动物实验的必要性，无其它试验方法可替代）：

实验需要40只雄性Wistar大鼠(180g-200g)，购自上海中医药大学动物实验中心。实验期间动物在SPF级条件下喂养。造模第一天，大鼠随机分为正常组(n=10)和模型组(n=30)，模型组以2mL/kg剂量于每周前3天连续腹腔注射0.5%的DMN溶液，正常对照组腹腔注射等量的生理盐水，共4周。2周末，随机取正常组3只，模型组6只做用药前观察，其余模型组大鼠随机分为模型组(n=12)和黄芪汤组(n=12)。接下来的两周，模型组和黄芪汤组每天分别灌胃生理盐水和黄芪汤1mL/100g。4周末，腹主动脉取血，脱颈椎法处死，摘取肝脏组织进行后续实验。(因为实验需要肝纤维化模型，临床实验无法操作，故选用大鼠进行实验。)

实验人员是否经过实验动物有关法律法规的培训？ ☑是 □否

实验人员是否了解在实验动物中心做动物实验的规章制度？ ☑是 □否

实验人员是否掌握了本次试验的有关动物试验操作技术？ ☑是 □否

列出本次试验减轻动物痛苦的措施。

本次实验采用脱颈椎法处死大鼠，大鼠尸体交由上海中医药大学动物实验中心统一处理。

课题负责人遵守实验动物福利伦理原则的声明

本课题组郑重承诺：本动物实验方法和目的符合人类的道德伦理标准和国际惯例，在动物试验期间遵守有关的法规、实验动物伦理福利原则和实验动物中心的规章制度。

课题负责人：[illegible]

日　　期：2011.9.10

动物实验伦理委员会审查意见：

批准实施

审查人：[illegible]

日　期：

上海中医药大学 实验动物伦理委员会
